
JOURNAL INFORMATION
==============================
NLM Title Abbreviation: Med Chir Rev
NLM Journal ID: 0034617

The Medico-Chirurgical Review

Publisher: Biomedical Journal Digitization Project

ARTICLE INFORMATION
==============================
PMCID: PMC4966179
PMID: 29917602
Article version: 1
Subjects: Articles

Varieties

Print publication date: 1828 Jul 1
Volume: 9
Issue: 17
First page: 217
Last page: 240
1828 May 24
License: This work is free of known copyright restrictions. For more information, please see PMC Back Issue Digitization.
License URL: https://creativecommons.org/publicdomain/mark/1.0/

==============================

69. GASTROLOGY—CORNARO—LONGEVITY.

There are three important personages in this world, who are in daily and hourly request by all classes of society—the cook, the doctor, and the undertaker. We have put the cook at the *top* of the list; for although he and the undertaker carry on many of their grand operations *underground*, while the doctor moves in higher circles, yet, as we love to trace things to their sources—and as we believe the cook to be full as much the cause of the doctor as the doctor is the cause of the undertaker, so we have constructed the order of precedence in the above manner. Perhaps a secret motive may have influenced us, also, on this occasion. It is not to be concealed, though with shame we state it, that the doctor, forgetting that he owes his very existence to the cook, has lately endeavoured to take the trade out of his benefactor's hands, and thus monopolise both branches of the profession. Whether the public are to be gainers by the doctors turning cooks, we shall not attempt to decide. It is probable that if the undertaker set up the trade of physic in conjunction with his own—that is, if he opened a shop for the sale of drugs next door to that where he manufactured coffins, people would be rather shy in *going* to the former, lest they should be *carried* to the latter. That there was a time when the faculty knew their own interest better than to quarrel with the cooks, is made evident by that sagacious and facetious medical historian, Mr. Wadd. He informs us that a celebrated modern physician always accosted the cooks of his patients in the following terms: "My good friends, accept my best thanks for all the kind services you render us physicians. Were it not for you and your pleasing poisons, the faculty would soon find themselves inhabitants of the workhouse."*

Since that period, the said faculty have made war on the cooks, in two formidable columns—the one headed by Drs. Kitchener and Paris, who took possession, at once, of the stew-pans and gridirons, warning the public against swallowing any thing that was not *prepared* according to the analytical and synthetical *formulæ* which they had devised. This was bad enough; for the doctor was thenceforth MAITRE DE CUISINE, and the cook reduced to a SUB. Still he had something to do under the direction of his superior officer. But Messrs. Abernethy and Johnson nearly completed his ruin. They strenuously advised the public *to live without food, or nearly so*—and, as a necessary consequence, to discharge the cook!

It is said, we believe, in Holy Writ, that it is not that which goeth *into* the mouth that defileth the soul. Modern sceptics have questioned this dogma—and none more resolutely than Mr. Abernethy. He tells, and tells us truly, that "there is a direct communication (perhaps association would have been a better word) between the stomach and cerebrum —and that the disordered state of the one will produce a like state in the other."

"Suppose a man is a glutton, and overcharges his stomach with all the cursed mixtures which a vitiated appetite can invent, what can he expect but the constant production of an irritable material, from the fermentation of the vegetable matter, and from the animal matter becoming rancid? The product is quite sufficient to account for all the train of evils which are sure to happen to the nervous system."

"When patients apply to me," says Mr. A. "and I see that their complaints are chiefly of the nature I have been describing, I tell them that *I am no physician; and I offend them stoutly*, when I tell them that they have their health in their own keeping." If a man were to live like Cornaro, Mr. A. assures us that "he would be rewarded for it by a *long* and happy life." That many people would lead a more *happy* life than they do, if they adopted the system of Cornaro, we have no doubt; but that the general range of human existence would be materially *prolonged* thereby, we have much doubt. Individual constitutions differ so much, that Cornaro's quantum of *twelve ounces* of food in the 24 hours would kill as many as it would cure. The single instance of Cornaro's longevity proves nothing. Fifty instances might be adduced, where drunkards and gluttons arrived at a very old age. Parr was

* Comments on Corpulency.

by no means a very abstemious man, and yet he lived to the age of 144 years. The *rule* which a sane medical philosophy should lay down for diet, does not consist in the number of ounces which a man ought to eat; but in the quantity which he can digest, without inconvenience to the health. It would, therefore, be very beneficial to patient and practitioner, if the signs or criteria of too much or too little ingestion were carefully investigated, rather than the attempt to fix a determinate quantum of food for all people—an attempt as futile as that of laying down the exact length of men's shoes. The shoe which would cripple one man, by the growth of corns, would fall off another man's foot, from its largeness, while crossing the streets, among carriages and equestrians! So it is with food. The quantity which would make one man uncomfortable for many hours after dinner, would scarcely fill the corner of another man's stomach, and would render him a walking—perhaps a hanging skeleton in six months.

"The diet (says Mr. A.) I have taken the liberty to recommend to the public is Cornaro's, with a few conundrums of my own, as Dr. Franklin says.* I do not pretend to have adhered to such a diet as Cornaro did. Oh, no! I acknowledge myself to have been a sinner; and I remember *once* having been living irregularly, and having been taking butter and sauces and sweetmeats, and indulging a pampered appetite, things that turned acid and rancid on my stomach; I was seized with pain in my bowels and headache, and had a sore throat; and I had a friend of mine, *a physician*, to look into my throat, and there was a long discussion as to what sort of cynanche it was to be; one said one thing and *the second* another; but I smiled and said, if you do not know what it is I know what will cure it; so I took a dose of calomel and jalap, and *lived upon toast and water for about ten days*, and I got rid of my sore throat and fever together."

We do not dispute the propriety of the talented writer's restricting himself to toast and water during a fever. Perhaps if Mr. Abernethy had been weighed at the commencement and at the end of this period, it would have been found that he had lost at least a dozen of pounds in weight—and consequently his absorbent system was furnishing the general system with a pound of good animal food, or, at all events, animal fat, every day. But we do demur to the following scale of diet and drink which the worthy lecturer has now printed on slips of paper for his patients.

"The RULES OF DIET, IN DYSPEPTIC CASES may be thus stated in an abbreviated form :—

"1. The food should be of the most nourishing and readily digestible kind.

"2. The quantity taken at a meal should not be more than it is probable the stomach will perfectly digest.

"3. The meals should be taken at regular periods of six hours, three times a day: and when the stomach can digest very little food, they may be taken four times in the twenty-four hours.

"4. Every meal of food should be reduced to minute sub-division and pulpy consistence by mastication, or otherwise; and suffered to remain in the stomach unmixed with liquids, in expectation that it will be dissolved by the juices of the stomach.

"5. Drink should be taken *four hours after each meal;* allowing that time for its perfect digestion, and two hours for the conveyance of liquids from the stomach before the pulpy food be again received.

"6. The drink then taken should not contain fermentable substances. It should be boiled water; which may be

* "Cornaro was given over by his physicians at the age of thirty-five; he saw that there was not the least chance of recovery, if he continued to swallow the trash they were in the habit of giving him, and that there was no good in putting food into his stomach, if his stomach could not digest it. What did it do there? Why, it played the very devil with Cornaro's guts. 'So,' said he, 'I dropped the plan pursued by my physicians, and adopted a regimen of my own.' The principal beauty of Cornaro's life was the happy state of mind in which his continued temperance preserved him. He limited himself to twelve ounces of food for each day, which was of a nutritious kind, and no inducement could prevail on him to exceed it."—*Lecturer.*

flavoured with toast, or prevented from producing a qualmish state of stomach, by pouring it upon a trivial quantity of powdered ginger.

"It is not meant by these rules to debar persons from taking a *small teacupful of liquid* with breakfast, or a glass or two of wine with dinner, if it seems to promote the digestion of their food."

It will be obvious that there is a considerable discrepancy between Cornaro's diet, which Mr. A. says he recommends, and that which is in his printed code of instructions. The rule, too, of not drinking till *four hours* after dinner, is equally impracticable and unphilosophical. The sensation of *thirst* is to be attended to, whether at or after dinner. Drink should not be taken when there is no inclination for it; but when Nature calls for liquid, the call of Nature should not be disregarded, whatever may be the hour which the hand of the clock points to at the time.

---

### 70. New Speculations on the Spleen.

Every body knows how many theories have been broached upon the structure and uses of this provoking viscus. We do not, of course, intend to enumerate them here, nor indeed could we, even though like the poet, we were gifted with the

"Ora centum, ferrea vox."

M. Jules Arthraud, however, has put another speculation on the record, and promises to prove what he advances, in a Memoir to be shortly read before the Institute. It appears that M. Stranz, in the year 1817, made some microscopic observations on the spleen of an elephant, which died at the Jardin des Plantes, and was surprised to discover in it none of the distinctive characters of either the fibrous or vascular tissues. This gentleman opened several of the filaments (filets), many of them half a line in diameter, of which the organ was composed, and found them filled with a pulpy substance, precisely similar to that which forms the nerves. From these facts he concluded, in our opinion hastily enough, that the spleen was a large nervous plexus, connected with the ganglionic system! M. Stranz was unable to continue his speculations, but M. Jules Arthraud pursued them with unabated ardour, and has come to the following conclusions.

1mo. In vertebrated animals, the existence of the spleen is always in connexion with that of the nervous system.

2nd. The spleen in the human fœtus does not appear distinctly until the second month of pregnancy, the period at which the ganglionic system is discovered.

3tio. The spleen accompanies the nervous system in all the changes which it undergoes from age, the descent in the scale of the lower animals, and lusus naturæ.

4to. In the true cases of acephalous fœtuses, the spleen is invariably absent.

5to. The substances which especially stimulate the ganglionic nerves, act strongly on the spleen, which enlarges under their influence.

6to. The tissue of the spleen, which our author calls *nervous*, preserves in a bath of pure water the density it had previously acquired in a mixture of seven parts of water to one of nitric acid, whilst the vessels which enter into its composition, soften and grow putrid.

7to. A number of facts induce our author to lay to the account of alterations in the spleen, an order of intermittent fevers, considered by many excellent practitioners, as belonging to the neuroses.

In short M. Arthraud assures us that in his coming Memoir, he will satisfactorily prove that the spleen is nothing but an electrical apparatus, producing certain changes to be hereafter described in the circulating blood. We trust our enthusiastic author will succeed as completely as he anticipates, but, in this particular instance we fear we must be classed amongst the tribe, which Swift, we believe, so sarcastically describes, whose philosophy is—to doubt.—*Journal de Progrés.*

---

### 71. Spinal Irritation.

Under this term, for want of a better, Dr. Brown, Senior Physician to the Royal Infirmary of Glasgow, has published, in the second Number of the Glasgow Medical Journal, a very long and by no means uninteresting paper, on a peculiar affection of the spinal nerves, frequently met with in young females, and sometimes in grown women or even males, which has hitherto, we believe, remained unchronicled by the medical practitioner. The complaint is not generally of a serious nature in itself, but leads to nervous affections of a troublesome character, if long continued. The author warns the reader in limine, against confounding this complaint with certain spinal diseases, as lateral curvature, inflammation of the spine, &c.—diseases with which it may occasionally be complicated. The complaint in question, which our author refers to "increased irritability of some of the spinal nerves," will be understood best—at least in its more common form—by the statement of a case.

*Case.* "Miss C. aged 17 (September 1822), of a robust make, and apparently in good health, for more than a year has complained of pain, situated below the left mamma. This has been fixed to one spot for nearly the whole time. It is a gnawing bruised feeling, increased materially by fatigue of any kind; and, after fatigue, it is attended with restlessness. It is relieved by reclining in the horizontal posture. It is not sore to the touch. The complaint has been treated by a surgeon in the country as a case of rheumatism. She has been bled and blistered for it, but without any good effect; and at last it has occasioned so much anxiety in the minds of her relations, that she is brought to Glasgow, from a considerable distance, for the benefit of farther medical advice.

"On examining the spine, it is found to be perfectly of the natural shape and appearance; but when pressure is made on it, about the 7th or 8th dorsal vertebra, she complains of a considerable feeling of tenderness, amounting even to pain; and she finds that the uneasy sensation shoots forward exactly to the affected part of the breast. She had not paid any attention to this tender part of the spine; indeed, she had no idea that there was any thing faulty there, till her attention was called to it by the examination.

"After I saw her, she had a dose of physic; 10 leeches were applied to the pained part of the spine, followed afterwards by a small blister; and the horizontal posture was enjoined. She was nearly free of pain in a few days, and returned home, with directions to repeat the blister, and to avoid fatigue."

Dr. Brown could bring forward 50 cases of a similar nature, from the Journals of the Lock Hospital, and from his private notes, but deems it unnecessary. He adverts merely to some *varieties* in those symptoms which attend the disease, when seated, as in the above case, in the lower part of the dorsal vertebræ.

"The site of the pain in the *breast* varies much. It is needless to mention, that it is occasionally in one side of the chest, occasionally in the other; but I am confident that it is much more frequently in the right than in the left. It is sometimes within a few inches of the spine, but much more frequently it is nearer to the sternum, and occasionally it is immediately under this bone. It is generally described to be a weary or bruised pain. It is seldom increased by the touch, but sometimes, though rarely, it is tender when pressed. It is usually, though not always, relieved or even removed by the horizontal posture.

"The tender part of the *spine*, on the other hand, in a great number of instances, is not attended to. It is not thought of till the affected part of the back is pressed, or till a sponge dipped in hot water be applied to it. In either case, a very sensible pain is felt, which, especially when the sponge is used, is occasionally acute and continues for some time. In general, pressing the spine not only occasions pain in that part, but the pain penetrates to the affected spot of the chest, thus distinctly proving the connexion between the two. It often happens that pressure on the spine occasions a feeling of oppression more than of pain in the chest.

"The pained part of the spine, in general, does not exceed an inch in diameter, though it occasionally happens that the uneasiness extends either above or below the tender part, appearing to radiate from it as from a centre, sometimes to a considerable distance. This pain of the side, excited by pressure, is almost in every instance felt on the same side of the spine as the pain in the chest. That is, if the patient have pain in the left side of the chest, then the left side of the spinous process of the vertebra is more tender than the right, and *vice versâ.*"

There is seldom any fever with these symptoms—cough is not often present—though sometimes it is convulsive, noisy, and unattended by expectoration. In a girl in the Lock-Hospital, there was pain on the left side of the sternum, as if the part had been bruised. This was distinctly connected with a tender state of the spine, below the middle of the dorsal vertebræ. In two other cases, there was, along with tenderness of the spine, an extended superficial sense of rawness and pain over one side of the thorax, extending even to the abdomen. It required a month's horizontal posture and an issue in the back. According to our author's experience, the source of the complaint is more frequently situated about the 8th or 9th dorsal vertebra than elsewhere.

Next, in frequency, to the middle of the back, this disease locates itself in the upper part of the neck, about the 2d or 3d cervical vertebra. In one very complicated case, there appeared to be two sets of symptoms—each of which radiated from a tender part of the spine, as if from a centre. There was pain in the left side, and numbness and pain in the left arm and leg, distinctly connected with tenderness situated about the lower dorsal vertebræ. After a time, a similar state of the 2d or 3d cervical vertebra took place, occasioning pain in the left side of the neck, commencing near the angle of the jaw, and extending upwards from the neck to the back part of the head, and even to the fore-head, following the ramification of the 2d or 3d cervical nerve. These two sets of symptoms were sometimes entangled and blended together. Posture was tried—then issues were opened on each side of the pained part. After three months' confinement the pains had disappeared; and the lady was allowed to sit up; but the pains returned, and she was again placed recumbent. The malady at length was merged in phthisis, which put an end to her sufferings. We are unable to follow Dr. Brown through all the cases he has adduced. These affections appear to him divisible into two classes—"those in which there is merely a morbid sensibility of a single nerve—and those in which there is a more general and constitutional irritability; in which the irritation is apt to affect different parts of the spine in succession, occasioning a train of singular symptoms. Dr. B. has rarely seen the pain in both sides at the same time. The following are the deductions or conclusions to which our author seems to have come.

"These are—that the immediate cause of the pain of the back and breast is spasm of one or other of the muscles, arranged along the spine, altering the position of the vertebræ, or otherwise compressing the nerves as they issue from the spinal marrow.

"That this spasm, in many instances, is strictly a *local* disease produced by fatigue, wrong posture, or other causes, and quite unconnected with the state of the brain, spinal marrow, or nervous system in general.

"But that, in other formidable instances, this partial, spasmodic, or wrong action of these muscles, is owing to a faulty state, perhaps an enlargement of the vessels of the brain, or spinal marrow. This state of the brain, as in many other diseases, gives rise to spasm, or even to convulsion of certain muscles; which partial symptom from its severity attracts the chief attention. This local affection is confined to those portions of the spine where there is the greatest motion, and where of course the muscles having the greatest activity, are most liable to deranged action or spasm. I imagine that this view of the subject is illustrated, and perhaps confirmed by various symptoms, which were observed in the different cases, and which without it, were very incomprehensible. The partial palsy, the affection of the sight, the giddiness of the head (for I find that this was a prominent symptom in several cases, especially in that of A. S.), all give some confirmation to the notion that the brain is affected in these severe cases.

"I have only to add farther, that if we pay attention to the number of the muscles, arranged along the spine, and to their functions, we shall see some reason for their being peculiarly liable to spasm. The variety of separate muscles in this situation is very great; and it ought to be kept in mind, that these are more constantly active than any other muscles, except the involuntary ones, since they are in a state of action in preserving the body in an erect state, as well as in every motion of the trunk.

"This state of the muscles, as being the immediate cause of the pain, and of various uneasy feelings, is certainly entitled to marked attention, and we find it much moderated by posture, by local application, and especially by friction; but my conviction now is, that we ought to direct our attention at the same time to the state of the brain, as being the source on which severe cases of this description depend."

With all due deference to the ingenious author, we should be inclined to refer the primary source of irritation to the uterus or digestive organs. In many cases, we have distinctly traced it to such sources —and not only these particular pains, but many others, in different and distant parts of the body. We would, therefore, recommend the practitioner to direct his remedial agents, in the first instance, at all events, to the organs alluded to; and, if he fail, he may then direct them to the brain. We do not, of course, take into account those cases where there is actual inflammation, or other disease, of the spinal column or its contents. These will require a very different treatment, the principles of which are now pretty well understood.

---

72. POISONING BY SULPHURIC ACID.

In the month of October, 1826, a young woman attempted to swallow three ounces of the sulphuric acid of the shops. The greater part of the acid was, however, rejected, and, according to her own account, only two or three spoonfuls went fairly down her throat. Her vomitings, cries, and convulsions, attracted the neighbours, who endeavoured to give her water to drink, but in vain, as she could not swallow. In the evening a physician was called to her assistance, who recommended leeches to the throat, and fomentations to the epigastrium; but the one produced no relief, and the other was insupportable. The wretched patient suffered inexpressible torture, and could only swallow a few spoonfuls of drink. It was on the fourth day from the accident that Dr. Lebidois saw the young woman. She was lying on her back, the lower limbs rigid, face pale, countenance sunk, tongue soft, moist, and white, as was the palatine arch; but the uvula and velum pendulum were of a deep red colour. She complained of severe pain in the tract of the œsophagus, augmented by pressure, or any attempt to swallow, cough, or even speak. The epigastrium was extremely sensible to pressure. The ingestion of the smallest quantity of fluids into the stomach caused nausea, and efforts to vomit. The abdomen was soft, and of natural temperature, pulse feeble, respiration slow, the lower extremities cold, intellect unaffected. In the course of the following days the symptoms became mitigated, and she could swallow some spoonfuls of broth; but emaciation advanced rapidly, without any febrile movements in the system. A troublesome cough now came on, and she sunk on the 15th day from the accident.

On dissection, the internal surface of the œsophagus was of a cherry redness, and its structure so soft, that it was easily lacerated by the fingers. In the stomach there were various appearances, as long stripes, of a deep red colour—several spots where the mucous membrane was destroyed, and red granulations sprouting up. Near the pylorus, there was a grey membraniform layer, like that which covers an old blistered surface, beneath which, the mucous tisue was of a vivid red colour. The small intestines were of a violet colour, contracted, and containing only bile and mucus. The large intestines were empty, and rigidly contracted. The heart was very large—nearly twice its natural size—marks of engorgement and inflammation in the lungs—nothing extraordinary in any of the other viscera.

The principal object which Dr. Lebidois appears to have in view, in the publication of this case, is to shew the remarkable want of febrile symptoms in a most intense gastro-enteritis. If, as the school of Broussais maintains, the phenomena of fever be owing to irritation or inflammation of the mucous membrane of the stomach and bowels, how is it that we had none of these phenomena present when the said membrane was intensely inflamed, and, in many places, destroyed? But then it is to be recollected, that inflammation of the stomach and bowels, resulting from general causes, as those of fever, applied to the constitution at large, is a very different thing from that phlogosis, which results from a direct local application of irritating or poisonous substances to the stomach itself. In the first case, the morbific agents (say marsh miasma) have acted on the nervous system, and reached the point of producing inflammation of the gastro-enteritic lining—in the other case, the local irritation acts at once on the stomach, and may not produce great constitutional disturbance in particular individuals.—*Archives.*

Several instances analogous to the above are on record, upon the authority of Tartra and others, shewing how disproportionate the general or sympathetic affection is to the local.

---

73. ON AN INTERNAL INFLAMMATION OF THE EYE, FOLLOWING TYPHUS FEVER. By ARTHUR JACOB, M.D. Professor of Anatomy, &c.

In the volume of Transactions just published by the Dublin Association, there is an interesting paper on the above subject, the principal particulars of which we shall lay before our readers.

That iritis is one of the sequelæ, or consequences, of the late fevers in Ireland, was first shewn by Mr. Hewson, in his Essay on Venereal Ophthalmia, and the fact is now fully established by the experience of Irish practitioners. Dr. Jacob himself has met with 70 or 80 examples in the course of one year. He is unwilling to apply the term iritis to this affection, because he does not think that the iris is the part primarily or exclusively attacked. All the internal structures of the eye appear to participate in the inflammation—especially the retina. In all the cases which our author has seen, the patients had suffered from previous fever, within the period of a few months. This affection occurs more frequently in young, than in old persons—it is also much more common among the poor than among the rich—and generally made its appearance about six weeks after the occurrence of fever. Both eyes were seldom affected. The inflammatory symptoms were usually preceded by those defects in vision which are considered to arise from disease of the retina, such as muscæ volitantes, clouds, luminous coils or stars. These, in general, had not existed for more than six or eight days before the appearance of inflammation—in some cases, however, they had existed for two months. The inflammatory stage was distinguished by the increased vascularity, the cloudiness of the transparent parts, alterations in the condition of the iris, pain, intolerance of light, lachrymation, and defect of vision. The vascularity produces the same appearances which have been observed in other forms of internal inflammation and iritis.

"In this inflammation the transparent parts are rendered more or less clouded or opake; the cornea especially has its margin or circumference almost always of a whitish or grey appearance, presenting an opake circle resembling the *arcus senilis.* The anterior chamber of the eye appears clouded, independent of the opacity of the cornea, arising probably from thickening of the membrane of the aqueous humor; this cloudiness is sometimes general, sometimes it presents a muddy patch behind the cornea, as in syphilitic iritis. In the worst form of the disease the lens itself becomes partially opake, reflecting light falling obliquely upon it, and presenting an opaline amber colour; indeed it is in this way I have observed vision to be destroyed where the disease has been fatal to the organ. The hyaloid membrane of the vitreous humor may possibly participate in this opacity.

"The iris is always altered in colour, the brilliancy of its tints being totally lost; it never, however, acquires the decided yellowish green observed in syphilitic iritis; neither have I observed the abscesses or tubercles, usually called globules of lymph, which characterize that form of inflammation; purulent matter is however occasionally secreted in the anterior chamber of the aqueous humor, constituting hypopion, or unguis. I have not observed that the secretion of purulent matter was a consequence of more intense inflammation; on the contrary, I have seen it in very mild cases, and even where the pupil contracted on exposure to light. The pupil is generally slightly irregular, but I have not observed that it contracts adhesions to the capsule of the lens, or that it becomes closed as in iritis; in many well-marked cases I have found it contract on exposure to light with considerable activity."

The patients generally complain of a stinging or aching pain darting to the temple or nose—but in many cases there is no suffering. Intolerance of light is almost always present, attended with scalding lachrymation. Vision is much impaired.

The treatment is not difficult.

"Bleeding, locally or generally, in proportion to the urgency of the symptoms; blistering, where there is much pain or intolerance of light; purgatives, antimonial medicines, and opiate stupes, are obvious means of relief. I am in the habit of using the extract of belladonna very freely, not only in this form of inflammation, but in every other, and more especially in syphilitic iritis, where there is so great a tendency to closure of the pupil and adhesion of its margin to the capsule of the lens. In the contracted state of the pupil its margin, at least when inflamed, is in contact with the capsule; but when dilated it is altogether detached from it, and consequently cannot adhere. It must however be admitted that the iris, when much altered by inflammation, is not affected by the belladonna; but even under such circumstances its use is not attended with any disadvantage. But it is not from its effect on the pupil alone that the extract of belladonna should be applied; very decided relief is obtained from its application in those cases where there is deep-seated pain, extending from the eye-ball to the temple, and especially in rheumatic inflammation. It may be used as recommended by Beer, in the form of ointment, rubbed in upon the temple, (half a drachm of the extract, with an equal quantity of mercurial ointment every night,) or the extract alone, softened with water, and daubed over the lids and brow, and kept moist for one or two hours with a light fold of old linen wetted every ten minutes, may be preferred. I do not, however, by any means recommend that the surgeon should depend upon these remedies alone; on the contrary, I believe that they will prove ineffectual in the majority of cases, and therefore we must have recourse to mercury, which has been found so valuable a resource in other cases. In my own practice I have found the relief from the use of mercury so certain and decisive, that I have trusted to it almost exclusively, with the assistance of the belladonna. I have generally found that two grains of calomel with a quarter of a grain of opium, three times a day, answered every purpose; and in the majority of cases I produced the necessary mercurial action, as marked by tenderness of the gums, in eight or ten days, by the use of three, four, or five grains of blue pill alone, three times a day; and if the pain should be severe, combining hyosciamus or belladonna with the dose taken at bed-time."

The sulphate of quinine has been sometimes used by other practitioners; but our author did not find it efficacious *without* mercury—and *with* that mineral it was unnecessary.

---

### 74. Paracentesis Abdominis through the Bladder.

Surgeons have an extraordinary dread of allowing the aperture in the peritoneum through which the fluid of dropsy is discharged, to remain open. They dread peritoneal inflammation. We have seen three instances where the water kept dribbling away for weeks, and even for months, without any inconvenience—nay, with positive benefit. In two cases the dropsy never returned so as to require an operation.

Dr. Andrew Buchanan, of Glasgow, has lately performed the paracentesis through the fundus of the bladder, with the view of leaving a permanent drain for the fluid collecting in the peritoneal cavity. The operation is, we believe, original, though something like it has been performed long ago by Mr. Watson, who, finding the vagina forming an external protrusion from the pressure of the fluid above, introduced the trocar into the abdomen, in that situation, and drew off the fluid. He informs us, that he practised this three times successfully. A surgeon in this metropolis has, we understand, operated in this way on an ovarian dropsy, but the result has not been yet ascertained by us. The female on whom Dr. B. operated, was not a good subject, being a pauper, who had been tapped twice before the present plan was tried, and whose spleen was enlarged and liver tuberculated. The instrument employed was the curved trocar, used for puncturing the bladder in cases of retention of urine. The canula was first introduced along the urethra, and the point of it carried towards the upper, and fore part of the bladder, pushing it as far up as possible, so as to put the coats of the bladder on the stretch. The stilette was then passed along the canula, and made to pierce the bladder, which it did with such ease, that no sensible resistance was experienced. On withdrawing the stilette, the water flowed in a full stream, till about 28 pints were evacuated. After withdrawing the canula, the urine flowed more copiously than usual, for a day or two, but then fell back to the scanty quantity generally secreted. No material inconvenience, however, followed this new operation. It was repeated three times afterwards, at various intervals, and never did any harm. At length the patient was worn out by visceral disease, and repeated accumulations of water. When examined after death, the liver was found tuberculated, and diminished in size—the spleen enlarged—the peritoneum free from inflammation—the fundus of the bladder presenting the marks of puncture, the most recent resembling a leech-bite, without any surrounding swelling or redness. The attempt was certainly ingenious, and could Dr. Buchanan have succeeded in establishing a fistulous communication between the bladder and the peritoneal cavity, his object would have been attained, though, under existing circumstances, this patient's life could not have been ultimately saved. The case in question proves that we have no reason to dread an effusion of urine from the bladder into the peritoneal cavity, after paracentesis in this place. And if this be the case, we imagine it must be granted, that there is equally little danger of air escaping into the abdomen, in consequence of leaving open the puncture of the trocar in common paracentesis abdominis. At the same time, we would use the precaution of proper bandaging, to keep the abdominal viscera compressed.

---

75. Clinical Report of the Hôtel Dieu. By M. Breschet.*

The subject of this Report is the affection called ranula, which is generally imagined to consist in an accumulation of saliva in the sub-lingual or sub-maxillary ducts, in consequence of their orifice having been choked up. The disease appears to have been tolerably well known to the ancient writers, at least Hippocrates and Celsus make mention of a tumour occurring under the tongue, though they entertained erroneous notions of its pathology. It has been observed by some writers that ranula is more frequent in infants than adults, but M. Breschet is of opinion, from several dissections, that common serous cysts beneath the tongues of children have often been mistaken for this particular disease. The viscidity of the saliva which has been considered as the cause of the disease, M. Breschet looks on merely as the consequence of the plugging up of the salivary canal, which may arise either from inflammation of the mucous membrane, aphthæ, or ulcerations in the canal itself. In dividing the frænum linguæ, some of the excretory ducts which open on its sides may be wounded by the knife, and afterwards become obliterated by the cicatrix; small calculi will also occasionally form within the ducts and check the flow of the saliva, a circumstance which happened to the late Mr. Cline, and gave him considerable inconvenience. The amount of these concretions is at times considerable, and the fluid, instead of resembling saliva, may be puriform, or even entirely purulent.

The symptoms of ranula are easily distinguished by a surgeon at all cognizant of the affection. The tumour is soft, whitish, regularly round or oblong, situated just beneath the tongue, having neither pain nor redness, nor, in fact, any of the characters of inflammation; elastic, fluctuating, at first so small as scarcely to be felt, and slowly acquiring size. In general, its volume is little larger than a nut or pigeon's egg, but in some instances it far exceeds this, and M. Breschet remembers having seen a man in whom the tumour appeared beneath the chin, and stretched from that in front of the neck, almost to the sternum, entirely preventing circulation. In a case which happened to Le Clerc, the tumour filled the mouth, thrust forward the teeth, and formed a prominence externally as large as a duck's egg. It may

* Repertoire, 1828.

perhaps admit of doubt whether these large tumours are really dilatations of the sub-maxillary or sub-lingual ducts, but, whatever they may be, they cause extreme distortion, doubling back the tongue, displacing or pushing out the teeth, altering the voice or preventing articulation altogether, and hindering suction in children, mastication and deglutition in adults.

The treatment resolves itself into palliative, and that which aims at a radical cure. The first consists in merely making an opening into the tumour within the mouth, and evacuating the fluid which it contains. Some have made their incision beneath the angle of the jaw, instead of within the mouth; but this plan is attended with danger of a salivary fistula. Le Clerc, however, operated in this manner with complete success; but probably his case was one of those where a large cyst exists filled with serum, instead of the true ranulary tumour. When a simple puncture has been made, the orifice quickly heals, the fluid again collects, and a fresh operation is required; indeed Petit relates an instance, where it was repeated ten times, and was ineffectual after all. Some have practised large incisions in the sac, others have cut out portions of it, and introduced into the opening tents of various kinds, portions of bougie, &c. while Sabatier employed a canula, which he left until the edges of the opening had become completely callous; but all these plans have failed in other hands. A large portion of the walls of the tumour has been removed, but though this operation may delay cicatrization, it does no more—the wound ultimately heals, and the malady returns. It has been proposed to extirpate the tumour, but unless the gland which fed it were extirpated too, the operation would in all probability be ineffectual, and it would be a bold attempt indeed to perform so severe an operation for so harmless a disease. If, as is supposed, the disease consists in an obliteration or obstruction of the salivary duct, then the employment of stimulant injections, as has been recommended, must be worse than useless, because they tend, of course, to increase the agglutination and obstruction. This is not the case when it is a common cyst, containing serum or lymph, for here the puncture of the tumour and employment of irritating injections would probably be of service.

The use of the actual cautery as a mean of opening the tumour, and preventing the opening from closing afterwards, is of very ancient date, as ancient indeed as the times of Hippocrates, who employed it himself. Acids have also been had recourse to, and Camper employed the lapis infernalis, although he confesses that it was frequently ineffectual; in short, all the methods which have been proposed or executed have been more or less unsuccessful in the long run. This want of success depends upon the tendency of the artificial opening, whether it be made by incision, or by the cautery, to contract and close; but M. Breschet tells us that Baron Dupuytren has at length discovered a method by which the closure of the wound is entirely prevented. Having made an opening into the tumour, the Baron takes a small instrument invented for the purpose, introduces it, and allows it to remain, just as the stilette is allowed to lie in the lachrymal canal, after the operation for fistula lachrymalis. This instrument appears from the description to be very similar in principle to that invented by Mr. Weiss for perforation of the soft palate. It may be made of silver, gold, or platina, and consists of a hollow cylinder, about three lines in length, and one, or one and a half in diameter, with a small, elliptical plate, convex externally, attached to either extremity of the tube. The use of these two small plates is to prevent the tube from slipping either *into* the dilated duct, or *out* of that into the mouth, for the opening having been made, and the tube introduced, one plate of course lies on the *inside* of the wall of the tumour, and the other on the *outside*, retaining the instrument in its position. If the tumour be of great size, or its walls much thickened, a free incision should be had recourse to before the application of the tube; in some cases it may be even necessary to cut out a portion, and allow the wound to nearly close prior to the introduction, but with these precautions our author affirms that M. Dupuytren has experienced complete success. Five cases are given in illustration, all of them occurring at the Hôtel Dieu, and therefore above suspicion of inaccuracy or colouring.

*Case* 1. Bruno Duchâteau, æt. 24, ex-tambour of the garde-impériale, was admitted, October 14th, 1807, with a small oblong tumour beneath the tongue, appearing to be a dilatation of the excretory duct of the submaxillary gland. Every kind of treatment had been adopted —incision—excision—cauterization, but all with the same success, or rather want of it. The Baron having opened the tumour with curved scissors, and given vent to a quantity of limpid, inodorous fluid, took up the little silver tube with a pair of forceps, and introduced it into the opening, so that one plate lay within the tumour, and the other on its outside, in the mouth. The disease very quickly disappeared, and in fifteen days the patient left the hospital, being able to eat, speak, &c. with the most perfect ease.

In the second case, the tumour had existed for several years, was about the size of a pullet's egg, and was perfectly cured by the above means. In the third case, there were tumours, one on each side. M. Dupuytren, for experiment's sake, introduced the instrument into one, but contented himself with merely making an incision into the other. The former was cured, the latter not, when the Baron treated it in the same way, with the same success. The two other cases were equally fortunate, save that in the last a tumour re-appeared, almost on the site of the one which had been treated. It was found, however, to exist in the dilatation of another duct, and after being punctured was dispersed.

The "sub-lingual and sub-maxillary tissues" are subject to an inflammatory swelling, which may be mistaken by an unpractised surgeon for common ranula, but which requires a very different treatment. The tumour in this case appears suddenly, increases quickly, and is accompanied by tension, pain, and redness. The following is a case of this kind.

Mary Eugrot, æt. 21, having irregular menstruation, entered the Hôtel Dieu, May 20th, 1821, with a hard tumour, beneath the lower jaw, formed by the sub-maxillary gland. The enlargement had been present for upwards of six years, was about the size of a pigeon's egg, painful to the touch, and, on the least pressure, there was forced into the mouth a fluid, made up of mingled pus and saliva. On the other side, there had formed beneath the tongue, within the last six weeks, a hard elastic tumour, which prevented speech, and caused some difficulty of respiration and deglutition. Its redness, pain, and hardness, induced M. Dupuytren to think it depended upon inflammation of the sub-maxillary duct, and accordingly he applied leeches, emollients, and derivatives. In four and twenty hours there was a marked amelioration, and at the expiration of a week she left the hospital, without either pain or tumour.

We have thus given an exposé of the practice of that distinguished surgeon, M. Dupuytren, in a very troublesome affection. Whether the plan he has recommended and adopted will be equally successful in other hands we shall not pretend to say; but, at any rate, it is worth a trial, particularly at our hospitals. By-the-bye, we think our English reporters somewhat behind their French *confrères* as yet; at least as far as the *utile* is concerned. In the *dulce*, i. e. the transmutation of certain sheets of letter-press into the much abused and much loved "filthy lucre," we verily believe that our native "gentlemen of the press" have fairly won the day. The fact is this, that the French reporter, for the most part, takes up a certain point of practice, and illustrates it, as far as possible, by a variety of cases; whereas, the Englishman is too much in the habit of giving a solitary case or so, because it is "curious" or out of the way; in other words—good for nothing.

---

### 76. Aneurism of the Temporal Artery.

It is well known that after arteriotomy, an aneurismal tumour occasionally forms in the temporal artery, and is productive of considerable inconvenience. In this country, the operation of opening the vessel is considered as exceedingly trifling, and constantly performed, but it would appear from a memoir on the subject by M. Desruelles in the Transactions of the Medical Society of Emulation, of Paris, that it has rather fallen into disuse in France. M. Desruelles, is a very warm advocate of the operation, and exclaims most eloquently against the reality of those dangers which alarmists have conjured up. According to M. D. indeed, the opening of the vessel is so simple, that if patients were more *accustomed* to it, they would certainly prefer it to venesection, like the eels we suppose, who, are so *used* to the process of skinning, that they scarcely feel it!

Several cases of aneurismal tumour are detailed by our author, in order to prove the facility of treating them, especially in the early stages.

*Case.* A soldier of the Royal Guard entered the hospital of Gros-Caillon, labouring under intolerable pains in the head. These were entirely removed by opening the temporal artery, and on the second day the bandage was taken from the vessel. Three days after this a pulsating tumour, about the volume of a pea, was perceived over the vessel, and in the course of a few days more it had perceptibly increased in size. Compression was employed, and at the expiration of a fortnight, only a small hard body could be felt, the pulsations having entirely disappeared.

The second case is that of a gentleman of full habit and bloated aspect who was bled by our author in the temporal artery. The vessel was not fairly cut across, and on the evening of the third day the patient felt something give way in the temple, where an aneurismal tumour made its appearance. Compression on the trunk of the temporal was completely successful in removing the disease. However, pressure is not in all cases attended with success, nor is the complete division of the artery a safeguard against the occurrence of an aneurism.

*Case.* M. V. an officer in the guards was bled from the temporal artery, for an obstinate cephalitis. The vessel was completely cut across, but the compression was slight, and thirty hours afterwards the bandage was entirely removed. On the third day the wound was painful, and on the fourth a small tumour formed. Compression was employed, but the aneurism increased in volume, and slight suppuration took place about the wound. Pressure on the temporal branch was then employed, but in spite of it, a violent hæmorrhage took place. Pressure was made upon the wound, and a good deal of swelling about the parts ensued. In the course of a couple of days the hæmorrhage returned, and M. Larrey tried ineffectually to discover the ends of the artery and tie them. The patient was bled, and the hæmorrhage ultimately stopped by the application of a cold poultice to the wound and ice to the head.

The fourth case was that of a young lady, on whom compression was tried by means of a ribbon, and almost entirely succeeded. She went to a ball, danced too much, the tumour re-appeared, and in a few days burst. M. D. tied the vessel an inch below the wound, and the disease was cured. From these cases our author thinks that aneurism of the temporal artery is owing either to the vessel not being cut across, or to an imperfect employment of compression.

He also thinks that when the disease has shewn itself, compression will be sufficient to effect a cure, provided it be properly and immediately employed. When this has failed, ligature of the vessel which feeds the tumour is to be resorted to, but the surgeon should never think of endeavouring to secure the two ends of the vessel, as they are engaged in the inflammation, and extremely difficult to find.

---

### 77. Regulation of Lunatic Asylums.

Most people in this country are aware (too many of them to their cost) how easy it is to legislate in the closet; but how difficult it is to adapt the regulations of the legislature to the practical purposes of real life. It is very common to find laws, which appear to be the very beau ideal of wisdom and justice, in the senate, turn out, in actual experience, to be, not merely lame and impotent, but positively tyrannical and unjust. Such is the difference between theory and practice! Upon the Bill now passing through Parliament, for the Regulation of Mad-houses, a gentleman of considerable talents and good practical observation, has published some strictures, which we shall notice cursorily in this article.

He observes that, since 1815, when a report was made on the actual condition of lunatic asyla, very great improvement has been made in private establishments, and men of education and high respectability have embarked large property, as well as superior skill in their construction and direction. The present bill appears to be badly calculated to encourage such speculations.

"Far from encouraging medical men to study the treatment of insanity, and to undertake the care of lunatics, as suggested by the Committee of the House of Commons in 1815, the Bill, as it is now arranged and presented to the House of Peers, tends to debase the whole profession; for it betrays a total want of confidence in their moral and medical character; so much so, indeed, that if the Bill pass into a law, no medical man who has any regard to his professional reputation can undertake the care of lunatic patients, either as proprietor or superintendent of any house for their reception, or as a regular attendant on the patients in it.

"The consequence must necessarily be, that private houses for the reception of lunatics will pass from the hands of medical men, whose characters are at stake, and who are admitted to be most competent to the charge, to those whose sole object is lucre; and who, being unrestrained by the honourable feelings of men of education, will readily find means of evading the provisions of the Act, if they can thereby accomplish their end."

The writer observes, that the duties of the Commissioners, according to the present Bill, will require ten or twelve days for each quarterly visitation of the London district—and, consequently, that "no medical man, of mature judgment, and in actual practice, can make such a sacrifice of his time as this duty will require. It may be remarked, too, that the treatment of insane cases does not *properly* belong to *surgeons*." We confess that this last part of the passage appears to us rather futile—and as coming with a bad grace from the writer, whom we know to have been a *surgeon* almost the whole of his professional life. If, indeed, insanity is to be treated on *metaphysical* principles, then we grant that the graduate of Edinburgh, and still more the M.D. of Oxford or Cambridge, just launched from the stocks, and who, by the way, will have ample time for his quarterly visitations, is a more proper personage for Commissioner of Lunatics, than the SURGEON who has been plodding in the dissecting-room. But we apprehend that it is full late in the day to preach these doctrines.*

It appears that some of the details in the Bill will be productive of great inconvenience and expense to the proprietors of lunatic asyla, without any corresponding advantage. For these we must refer to the pamphlet. The following passage, however, we shall extract.

"The Commissioners for the London district, and the Justices of the Peace in counties, may refuse to grant or renew the annual license, *without assigning any reason*; and against such decision there is no appeal!

"As the law now stands, the Commissioners of the College of Physicians, and County Magistrates, are bound to grant licences.

"The proprietors of houses are perfectly ready to submit to any penalty commensurate with the offence which the legislature may think proper to impose. But they feel it to be an extreme hardship that, after devoting themselves for many years, perhaps solely, to one line of practice, and expending large sums of money—in many instances their entire property—in forming respectable establishments; both their reputation and property should be exposed to ruin by this arbitrary power being given to those who are likely to be swayed by ignorance of all that relates to the insane, or by local and popular prejudices.

"Licenses may be revoked also by the same authorities; and such refusal or revocation is forthwith to be published in

---

* The honorable and zealous Member of Parliament (Mr. Gordon) who is "working the Commission," stopped the writer of this article the other day in the street, and complained of the dilemma into which he had got with the DOCTORS, for recommending a proportion of *surgeons* to be put on the list of *lunatic* Commissioners. The writer, who is a professed enemy to all *monopolies*, advised Mr. Gordon to persevere strenuously in the *equation* of lunacy among both classes of the profession. The honorable M.P. seemed highly pleased at having the sanction of a critic for his proposed scheme.

the *London Gazette*, or a county newspaper. If, after such publication, the proprietor continues to keep two or more patients for fourteen days, he is declared guilty of a misdemeanour. No provision, however, is made for the disposal of the patients in such event. In many instances their friends reside on the Continent, or in the East or West Indies; or there is no immediate relation who will receive them, or they are wards of the Court of Chancery; and in that time perhaps the patient could not be disposed of. The proprietor of the house must either turn all these helpless beings adrift, or retain them at the risk of a prosecution."

There are many clauses in this Act which certainly appear to be very objectionable—for instance, nocturnal domiciliary visits from the Commissioners, which would be sure to create universal consternation and agitation among insane inmates, who are so susceptible to every impression.

"The *lunatics* themselves, too, are at liberty to require the assistance of any minister or spiritual teacher; and the reason for refusing it is to be registered! The patient, however, is the last person to whom such a power ought to be given; but when he learns that the law sanctions his request, it will be constantly preferred, and the refusal of it be a cause of prejudice against the superintendent, for whom every patient ought to possess great respect."

When there is religious monomania, which is by no means rare, such communications would certainly tend to increase the malady rather than diminish it—for every one knows that, to reason with the insane on the subject of their hallucination, is uniformly prejudicial to the patient.

The certificate is to be signed by two medical men, who shall have *separately* examined the party. This is considered a great hardship in the country, where "it frequently happens that there is but one medical man within fifteen or twenty miles." We wish the writer would point out these happy regions; and we will be bound that they shall very soon be peopled with a plentiful colony of the MEDICI family. The author observes that, "throughout the country, medical practitioners are generally rivals, and are very apt to espouse adverse views and opinions"—hence it would, he thinks, be very difficult to obtain their concurrence in the necessary certificate. We think this difficulty is magnified; although we cannot help agreeing with the author, that the certificate of one *regular practitioner* is sufficient, provided the patient be not sent to any establishment with which the certifier has a professional or pecuniary concern. The clause relating to medical attendance in private asylums is strongly objected to by the author, as inconsistent and impracticable.

"First, Why should thirty-one patients have the benefit of a daily medical visit, while thirty are to receive only six visits in a month? Second, Patients may be divided into two classes; those in whom the disease is of long standing, and whose recovery is nearly hopeless—and those whose disorder is of recent occurrence. In old-established houses, a large proportion of the patients usually consists of the first class. Their bodily health may be good, their mental disease subject to little or no variation. A register, containing a statement of what attention the medical attendant has bestowed on every such patient, with observations on his case, would be uselessly voluminous and burdensome. In most instances, such cases require no more than a glance of the physician's eye, or, at most a few words of conversation: to devote greater attention to them is inexpedient. No medical man of reputation will undertake to superintend or visit a licensed house, if he be compelled to enter daily all his reasons for his moral, as well as his medical treatment, in a register, which is to be examined in his absence by strangers, who may wholly misinterpret his views, and to whom he has no opportunity, even if he wished it, to give an explanation."

The last objection which we have room to notice, is the power granted to the Commissioners or Visiters to discharge patients. There can be no doubt that, in many instances, especially where the Commissioners or Visiters are not medical men, there must be much incompetency as to the decision respecting such discharge. A melancholy example is adduced, where a late President of the College of Physicians desired the discharge of two female patients—one of which drowned, and the other hanged herself, very soon afterwards ! ! The writer concludes thus :—" Finally—the bill is an experimental one for three years ; but if it pass, the evils flowing from it will be immediate, and many proprietors probably irretrievably ruined before that period expires." Believing that the framers of the Bill are actuated by the most disinterested and humane motives, we have little doubt that these observations of a man practically acquainted with all the details of a lunatic establishment, will have their proper influence, and may tend to modify some of the objectionable clauses, ere yet they pass into a law.

---

78. SENSATION—MOTION—VOLITION.

From a little memoir lately published by Magendie, (and translated into English*) on some recent discoveries relative to the functions of the nervous system, we have selected the following extract, as both curious and interesting.

" Undoubtedly, it would be of the utmost importance to ascertain how sensation and motion,—which, as it has just been stated, have their seat in the spinal marrow,—are propagated to the head, and extended to the brain and cerebellum ; or, to be more explicit, how the impressions perceived by the senses and the determinations of the will are transmitted to the spinal marrow. Here experimental difficulties become almost insuperable ; and I must confess that up to this day I have reaped nothing satisfactory on so delicate a question, and one which appears intimately connected with the most important secret of life.

" The innumerable experiments I have unsuccessfully tried hitherto, have, however, enabled me to ascertain one fact, which appears to me worthy of being noticed by physiologists, and respecting which, as far as my information goes, nothing yet has been advanced.

" If in a living animal you deprive the cerebral hemispheres of the power of acting, the animal will run straight forward with astonishing rapidity, as if propelled by some invisible and irresistible hand. If, on the contrary, the cerebellum be deprived of the power of acting locomotion assumes quite an opposite course —the animal recedes. It is a most remarkable phenomenon, for instance, to witness a bird, slightly wounded in the cerebellum, effect, for several successive days together, no other motions, either for walking, swimming, or flying, except in a retrograde direction.

" There should appear to result from these experiments, that any animal, otherwise enjoying its natural state of health, is placed, as it were, between two powers, which counteract and balance each other —the one impelling forward, the other backward ; and that these two powers are completely under the influence of the will.

" A disease of the horse, and which is not generally known, seems perfectly calculated to elucidate these last results. Farriers term it *immobility* ; and, in fact, if you attempt to drive the animal backward, let the means and strength you resort to be what they may, it remains rivetted to the spot,—the motions forward, on the contrary, are remarkably easy, and at times seem to be effected independent of the will.

" If the consequences I have just drawn be correct, the disease must consist of some physical alteration in the brain, or in some obstruction or other in the action of that organ.

" A few days ago I had two horses afflicted with immobility examined, and my conjecture was perfectly correct. In both, the brain was evidently diseased and the cerebellum perfectly sound.

" It then appears demonstrated, that these two opposite powers of the brain and of the cerebellum exist in animals ; and that in some peculiar cases these powers may resist the influence of the will.

" Is this the case with man ? Can our motions, which execute with so much precision the dictates of the will, cease to obey their commands, and be, as it were, in a state of rebellion ? Finally, is the faculty of volition distinct from that by which our motions are regulated ? Such are delicate questions we hardly dare to venture upon—they seem to lead to arduous abstractions, the insuperable limits of human understanding ; but,

* The translation is published by Mr. Nimmo, in the Borough.

however, I have witnessed myself, and I have had an opportunity of studying for several successive weeks, in a well-informed man, perfectly qualified for self-observation, a complete distinction between the will and that power by which our movements are regulated.

"Subsequent to violent grief, the person I allude to, to his utmost surprise, was suddenly deprived of the influence of his will over his motions; in spite of himself, he was compelled to assume the most ridiculous attitudes, and to make the most extravagant contortions. The eccentricity of his actions and postures baffle all description; in certain cases his motions would be natural: thus, without the slightest intervention of his will, he was seen to rise and walk precipitately forward, until he came in contact with some solid body that impeded his course;—at other times he would recede backward with equal promptitude, until he was checked by some similar cause. In other instances he was observed to recover the use of certain motions, and to remain incapable of directing others. It was thus that his hands and arms frequently obeyed the dictates of his will, and more frequently again could he regulate the muscles of his features, and those connected with the organs of speech. At times he was allowed to walk backward, whilst his progress forward became totally impossible; he then would resort to this mode of progression to reach the objects he had in view. This state lasted four calendar months, and terminated most successfully. A few grains of a substance which chemistry has lately discovered, (the sulphate of quinine,) sufficed to confine his motions to the immediate dictates of his will.

"It may then be correct to admit, that the faculty of volition is perfectly distinct from that faculty by which our motions are directed and classified into regular acts. Such is the only consequence I wished to infer from the fact I have just stated. Several others rush to the mind,—to follow them up would make a metaphysician of me, and I mean to remain a physiologist."

---

### 79. SUCCESSFUL LIGATURE OF THE INTERNAL ILIAC.

Another piece of bold surgery has been recently displayed by the transatlantics. Mr. White, of Hudson, has tied the internal iliac artery—with the solitary precedent before him of Mr. Stevens' operation, recorded in the Medico-Chirurgical Transactions. The patient, in the present instance, was a man aged 60, by trade a tailor, who presented a tumour about the size of a child's head, situated on the left hip, directly over the sciatic notch. It was of ten month's standing, and unattended with pain. The general health was good, with the exception of rheumatism.

"Upon making an examination of the tumour, the skin was found not discoloured, fluctuation was perceptible, but there was no pulsation. The absence of the last symptom rendered it difficult to decide upon the nature of the case, and accordingly we postponed giving an opinion until a consultation could be held with his family physician, Dr. HICKS, of Columbiaville. The doctor stated that the tumour could be removed by pressure, when small, but, from other cases he had seen of a similar kind, he presumed that it contained pus. He proposed opening it, and as that was the only way of ascertaining, unequivocally, the nature of the disease, we acquiesced. He accordingly punctured it, and nothing but florid blood made its appearance. A probe was passed in, and an aneurismal sac was found, about five inches deep. It was also discovered that the parietes of the sac were very firm and unyielding, which accounted for the absence of pulsation. After allowing a pint of blood to be discharged, the orifice was closed with a suture and adhesive plaster. It was observed that after this and also subsequent discharges of blood, the sac would fill again and the tumour resume its usual dimensions. The disease was supposed to be produced by repeated falls upon his left nates. He was a man of rather intemperate habits, and, when stupefied by liquor, would fall, as they expressed it, like a log. Being also lame in his left limb, from rheumatism, he, consequently, was most liable to fall upon his left nates."*

* Amer. Jour. of the Med. Sciences, No. 2.

A consultation was held, when it was unanimously agreed that the disease was aneurism, and the question arose whether it would be better to tie the gluteal or the internal iliac artery. The latter operation was determined upon, and performed in the following manner.

"On the 23d of October he was laid upon the table, and an incision was made of a semi-circular form, commencing two inches to the left of the umbilicus, and ending near the external ring. It was seven inches in length, and the convexity of it was towards the ileum.

"After dividing the skin, cellular substance, and superficial fascia, it became necessary to secure a few small arteries. The tendon of the external oblique being exposed, was next divided, and then the internal oblique and transversalis with its fascia. The peritoneum, which now presented, was detached from the iliacus internus and psoas magnus muscles, with the fingers, and was pressed with its contents towards the right hypochondriac region, by the assistance of my father. The external iliac was immediately felt, and by passing the finger towards the sacro-iliac symphysis, the internal iliac was distinctly recognized. The artery was then exposed with the handle of the scalpel, and the ligature passed under with the Philadelphia needle, one inch from the bifurcation. Instead, however, of drawing up the needle part with the hook, I found it more convenient to take it with the dressing forceps. One ligature being passed, it was found necessary, from the great depth of the parts, (being about five inches,) to pass down the knot with Dr. A. E. Hosack's knot applicator. The ligature was then firmly tied, and the parts were brought together with sutures and adhesive plaster.

"In this operation the same difficulty existed as in the case of ligature of the common iliac, by my distinguished friend, Dr. Mott, of New York, viz. the constant protrusion of peritoneum from abdominal compression created by the struggles of the patient.

"Some pain in the bowels, and fever came on a few days after the operation, which were removed by venesection and a laxative. Union by the first intention had taken place to a considerable extent at the first dressing on the eighth day. A considerable quantity of pus was discharged during the first four weeks, at the expiration of which time the ligature came away. The tumour has discharged its contents gradually, and the parts have assumed their natural appearance. The patient has so far recovered his usual state of health, as to be able to walk about his neighbourhood."

We need hardly say that this is another honourable trophy added to the surgical reputation of our American brethren. Verily these sons of the new world are not degenerating in their progress along the stream of time, like some of the worn-out monarchies of the old hemisphere.—*Perge!*

---

### 80. Thames Water.

The Commissioners, (Dr. Roget, Mr. Brande, and Mr. Telford,) have at length made their report, and, upon the whole, we consider it a proper one—such as men, unbiassed and unprejudiced, would naturally construct from the evidence that came before them, and their own reasoning on the subject. It is some years since we publicly remarked on the abominable practice of supplying a great portion of the inhabitants of this metropolis with water issuing from their own cloacæ! Who would believe that such a supply of water would have found venal advocates, even in the medical press of this country? Such, however, is the fact. The following extracts from the official report will be sufficient for our present purpose.

It appears that the quantity of water required for the daily supply of the inhabitants of the metropolis, and for the use of the various manufactories requiring it, amounts to about 29,000,000 gallons, or 4,650,000 cubic feet; the greater portion of which is derived from the Thames. The Report states, "that the present state of the supply of water in the metropolis is susceptible of and requires improvement; that many of the complaints respecting the quality of the water are well founded, and that it ought to be derived from other sources than those now resorted to, and guarded by such restrictions as shall at all times ensure its cleanliness and purity." With respect to the Thames water, the Report states that specimens taken from various parts of the river from whence the supply is obtained, had been put into the hands of Dr. Bostock, an eminent chemist, for the purpose of examination, and the following is an extract from the Report he presented on the subject:—

"It appears that the water of the Thames, when free from extraneous substances, is in a state of considerable purity, containing only a moderate quantity of saline contents, and those of a kind which cannot be supposed to render it unfit for domestic purposes, or to be injurious to the health. But as it approaches the metropolis, it becomes loaded with a quantity of filth, which renders it disgusting to the senses, and improper to be employed in the preparation of food. The greatest part of this additional matter appears to be only mechanically suspended in it, and separates by mere rest. It requires, however, a considerable length of time to allow of a complete separation, while, on account of its peculiar texture and comminuted state, it is disposed to be again diffused through the water by a slight degree of agitation, while the gradual accumulation of this matter in the reservoirs must obviously increase the unpleasant odour and flavour of the water, and promote its tendency to the putrid state."

"Regarding the greatest part of the impure matter in the Thames, as mechanically mixed in it, we may conceive that a variety of incidental circumstances will affect its quantity, in the same situation and under the same circumstances of the tide; but the observations are sufficiently uniform to warrant us in concluding that the water is in the purest state at low tide, and most loaded with extraneous matter at half ebb. It would appear, however, that a very considerable portion, if not the whole of this extraneous matter, may be removed by filtration through sand, and still more effectually by charcoal and sand. The Commissioners, however, say that they consider that the most perfect system of filtering can effect only a partial purification. It is stated that the Thames water, in what is called the London district, has suffered a gradual deterioration within the last ten or twelve years, to such an extent as to have almost destroyed the fishermen's trade between Putney-bridge and Greenwich." The Commissioners add, that "it is obvious that water receiving so large a proportion of foreign matters as we know find their way into the Thames, and so far impure as to destroy fish, cannot, even when clarified by filtration, be pronounced entirely free from the suspicion of general insalubrity. In reference also to this question, we apprehend that there are no grounds for assuming the probability of any improvement in the state of the water drawn from the London district of the river."

Water taken from the London Docks had been examined, and it did not contain the least quantity of copper. The Report says, the New River water is not open to the same objectionable impregnations as that of the Thames, but is susceptible of great improvement. It recommends the putting a stop to the draining the adjacent lands into the New River, and also the preventing persons bathing. It adds, "Great benefit would result if the inducement to bathe in the New River were superseded by the establishment of baths in the neighbourhood of the metropolis, to which the public might, under certain regulations be allowed access." It has been stated in evidence, that the New River Company have voluntarily offered to furnish sufficient supplies of water for a purpose of such manifest utility.

The Report does not enter into the examination of any practical schemes for a general and better supply of water to the metropolis.

There can be no remedy—no effectual remedy, for the evil, but transferring the source of supply to a part of the river beyond the reach of the tide—or to some other river, uncontaminated by common sewers.

---

### 81. DISEASES OF INFANTS.

In a clinical report which Dr. Guibert has made from the Hospital for Children, in Paris, several cases are detailed where tuberculation of the lungs, and even of other organs, had gone to a great extent, before the age of three or four years. These cases prove, we think, that tubercles, or at least their germs, are born with us, their more or less rapid development afterwards depending on various causes, as bad diet, cold, inflammation,

&c. We shall state the particulars of one or two cases.

*Case* 1. Eugene Godard, aged three years, had been for some time affected with cough and chronic diarrhœa. To these symptoms were added pain in the epigastrium, delirium, convulsions, and violent fever. He was received into the hospital in this state, and died three days afterwards.

*Dissection.* There were nearly five ounces of serous effusion in the brain—the membranes thickened and opake—and several portions of cerebral substance softened down to an almost purulent fluidity. In the chest, the right lung was found to be nearly in toto disorganized by hepatization and tuberculation, with several tuberculous excavations, such as are seen in confirmed phthisis of older subjects. The left lung was tuberculated in many places. The abdominal organs were sound.

*Case* 2. Louis Gelé, aged three years, had coughed for some months, and considerably emaciated. He had also diarrhœa, with constant fever, exasperated in paroxysms every evening. In this state he entered the hospital, on the 23d of January. The chest sounded badly—the emaciation advanced, and he died on the 2d of February.

*Dissection.* The arachnoid was thickened and opake, with albuminous exudation beneath—several portions of the right hemisphere of the brain were in a state of mollescence, resembling bloody bouillie. Both lungs were completely studded with tubercles, and, in many places, hepatized. There was a cavern in the right lung filled with tuberculous matter, that drained into it from broken-down tubercles in its neighbourhood. On the internal surface of the ventricles of the heart, there were tubercles, as also in the liver, spleen, and mesentery.

These cases, and a great many others which the author has brought forward, of a similar kind, ought to put practitioners on their guard, when called to children labouring under febrile or inflammatory excitement. The brain, the lungs, and other organs of infants, are nearly as prone to diseases as those of more adult age.

---

### 82. Hospital Practice.

### 1. *Fracture of the Cervix Humeri.**

Charles Bell, æt. 21, a robust sailor, whilst upon deck, fell with much force upon the shoulder, which deprived him, in a great measure, of the use of that arm. Nothing was done for him, and, six weeks afterwards, he entered Guy's Hospital, the shoulder being wasted, and the tubercles of the humerus not above the third of an inch from the acromion. On carrying the arm from the side, and rotating it, there was a crepitus perceptible, the tubercles moving *with* the shaft of the bone. On pressing just anterior or posterior to the "tubercles," there was acute pain, and if, at the same instant, the arm was rotated, crepitus was felt. The motions of flexion and extension were imperfect, abduction altogether gone, but he could still lift considerable weights. A pad was placed in the axilla, and the arm kept fixed to the side by proper bandages.

In a clinical lecture on the above case, Mr. Key remarked, that it sometimes requires a very accurate examination to detect fracture of the cervix humeri, on account of the little displacement which occurs. The limb, in these cases, is slightly shortened, and somewhat deformed—there is inability to raise the arm, though the underhand motions still remain—and, thirdly, if the arm is raised and rotated at the same time, there is a crepitus; whilst, if the arm be simply rotated, there is very frequently none. Sometimes the most careful examination will not detect this latter sign, as happened to Mr. K. in a case where the other symptoms of the fracture were distinctly present, although the crepitus was only accidentally discovered on the fourth day. When there exists considerable tumefaction, the crepitus is most readily detected at the coracoid process, which is explained by the circumstance, of bone being a better conductor of vibration than the swollen soft parts around the joints. It is laid down as a distinctive character of fractured cervix humeri, the preservation of the underhand motions of the arm, and the power of lifting heavy weights, "which can only be effected," says the reporter, "by the action of the biceps." If, by

* Med. Gaz. No. 22.

this, it is meant that, in lifting a heavy weight, the biceps only is in powerful contraction, we beg leave to differ from the reporter toto cœlo. If the reporter ever saw a man lift a considerable weight solely, or even *mainly*, by the action of the biceps, all we have to say is, that he has seen more than any body else ever saw before him. The thing is utterly impossible, and if the gentleman will only take the trouble to raise a fourteen-pound weight from the floor, he will quickly find it so. The action, so far from being performed entirely by the biceps, is performed almost entirely by those great muscles which arise from the sides of the chest and neck, and are inserted about the upper end of the humerus and scapula, as the trapezius and levator scapulæ, the pectorals, serratus, and latissimus dorsi. When the fracture is in the cervix humeri, it is *above* the insertion of the great pectoral and latissimus dorsi, as well as of the scapulary muscles which are inserted into the tubercles; consequently, these muscles can act with considerable force in raising the load: but we should like to see the man who could lift even a moderate weight when the fracture is *below* the insertion of these muscles, although the biceps still retains its power.

To return to the crepitus;—the circumstance of this being felt opposite the coracoid process, has led, in some cases, to the mistake of considering the case as one of fractured cervix scapulæ. When it is remembered, however, that this is so rare an accident, that Sir Astley Cooper never saw an instance of it, the error will not be likely to occur. It is singular that Mr. Abernethy, in his lectures, speaks of this fracture as if it were nothing unusual. He describes the whole joint as dropping down, "the cup and the ball and all, leaving a great vacancy under the acromion." The accident, according to Mr. A. is exceedingly liable to be mistaken for dislocation; but we think it not improbable that Mr. A. may have been himself mistaken,—at any rate, it would be satisfactory to learn in how many dissections the worthy lecturer has found the fracture where he describes it.

### 2. *Case shewing the Effects of Fear upon the Results of Operations.*

John Cooper, æt. 14, was admitted into St. George's Hospital, April 9th, 1828, under the care of Mr. Brodie, with extensive disease in the left tibia. The leg was somewhat swollen, the foot very much so, livid, and œdematous. In various parts of the leg, and opposite the inner ankle were ulcerated openings, leading to carious bone. There were some symptoms of disease within the ankle-joint, but they were slight and indistinct —there appeared, also, to be a slight sub-luxation of the head of the tibia backwards, on the condyles of the femur, although there was no sign whatever of disease in the knee. The disease had commenced suddenly five months previously, without any obvious cause, save that the boy had been in the habit of dabbling about with his feet in the water for some time before he was laid up. He was first attacked with pain on the inside of the ankle, increased on motion—then came swelling of the joint, and the ulcers over the tibia appeared at various intervals. He was ordered a generous diet and quinine, but there being little alteration by the 24th, the limb was on that day removed above the knee. On examining the amputated leg, it was found to present a good specimen of necrosis of the tibia, in several of its stages. The periosteum was red and thickened—new bone laid down to some extent—the old in some places unaffected, in others yellowish and opaque—in others, again, dead, and separating to form the future sequestra. The cartilages of the ankle-joint were destroyed by ulceration—the knee quite sound. On the same day on which this patient had his limb removed, another suffered amputation of the leg by the same surgeon, Mr. Brodie, and both, after the operation, were placed in the same ward, indeed, in opposite beds.

For the first few days, Cooper, whose case we are relating, went on but indifferently—there was great irritability—brown tongue—pulse at 120. On the 4th day, the stump was dressed, and had not a very promising appearance. The tinct. benz. c. was applied to it, and, on the 30th, (6th day) it looked somewhat better. From this time he certainly improved, having lost a good deal of his irritability, but the stump was a bad one, the flaps having retracted, and the bone sticking out. On the 2d May, he was ordered bark and full diet, and appeared to be doing well. On this day, however, or the next, the other patient who had been operated on, and lay in the opposite bed, died; and from this instant the most decided change was observed in Cooper. The irritability was increased tenfold—the pulse became small and extremely rapid—the countenance sunk and anxious—tongue dry, and reddish at the tip—mouth parched—bowels purged. He was ordered port wine, with sulphate of quinine in dilute sulphuric acid; tincture of orange peel, and tincture of opium. On the 7th, he appeared almost moribund, and was harrassed with a violent purging. Chalk mixture was ordered, and port wine, eggs and brandy, &c. given with a liberal hand. On the 8th, the purging had ceased, but the chalk mixture had been carried on, by mistake, to a much larger amount than was required. He could take no solid nourishment, and was in a very hopeless state. On the evening of the 11th, he died.

*Dissection.* Matter was found burrowing between the muscles of the stump, and running up in the cellular membrane surrounding the great vessels. The marks of recent inflammation were discernible in the chest, and superficial abscesses in the lung.

There being no trace of the thoracic inflammation during life, or at least the symptoms, if they did exist at all, being so obscure as to escape notice, shew that this was one of those cases of insidious inflammation of the lungs, which sometimes occur after great operations or injuries.

3. *Gangrene after simple Fracture of the Leg—Depletion employed—successful Issue of the Case.*

Henry Wake, æt. 53, was admitted into St. George's Hospital, May 1st, under the care of Mr. Brodie, with simple fracture of the left leg, about its middle. The tibia alone was broken, the fibula remaining sound, but there was another fracture into the knee-joint, which was also a simple one. The leg was put up in junks, and next day there was found to be much swelling and tension of the limb, together with discolouration of the integuments about the seat of the fracture, and vesications filled with bloody serum. The pulse was full and quick, but the limb was of its natural temperature, and the constitution very little affected. The junks were removed, and the leg laid out upon a pillow without either splint or bandage. Next day the vesications had increased. V. S. ad ℥x.—salines with sulphate of magnesia—cold lotions.

4th. Whole limb swollen up to near the groin, and of a yellow bilious tint—foot and lower part of leg œdematous—vesications very large and black—great discolouration. The pulse is full and has an extraordinary degree of force, but otherwise the constitution appears unaffected. Blood drawn yesterday a little buffed. On the 5th, he was much the same, and the bleeding was repeated, whilst the proportion of spirit in the lotions was increased. The blood drawn was cupped and buffed, but on the 6th he was considerably improved, the vesications being broken and collapsed—the discolouration fading. From this period the patient went on well.

This is an interesting case, first, as shewing that peculiar disposition to gangrene which sometimes follows injuries, and secondly, as proving the good effects of blood-letting in a case, in which, à priori, one would be disposed to dread its use. The patient, however, was of a bloated habit, and this, with the full and bounding pulse, were pretty fair indications of the treatment to be employed. The absence of constitutional symptoms throughout is certainly remarkable.

4. *Chronic Inflammation of the Testicle.*

It is well known that not many years ago it was the custom to remove testicles for an affection which is now cured daily by the use of mercury—we mean the chronic inflammation of the organ. The following is a case of this kind.

Thomas Nibbs, æt. 22, a healthy looking brickmaker, was admitted into St. George's Hospital, April 9th, 1828, under the care of Mr. Brodie.

In the course of last Summer, he says he fell into a well when in a state of intoxication, and next morning found the left testicle swollen and very painful. He was obliged to lie up for two or three days, but then, the swelling having in some measure subsided, he went to work again, and has gone on from that time, sometimes better and sometimes worse, up to the present period. He had a gonorrhœa three years ago, but had entirely recovered from it previous to his meeting with the accident. He has never had stricture, and always enjoyed good health.

The present symptoms are—left testicle nearly double its natural size, and uniformly hard, except at its anterior and outer part, where it is somewhat softer, and pressure gives the sensation, as if the testis itself were touched. There is, on the whole, little pain on pressure, though one point on the inner side is both more prominent and more sensible than the rest. The shape of the enlarged testicle is very much like that of the heart, but on the upper part, corresponding to the basis of the triangle, there is superimposed a globular swelling, having a distinct fluctuation, and apparently a collection of fluid in the upper part of the tunica vaginalis. It cannot be pressed up through the ring—the cord is healthy. April 11th, he was directed to take three grains of the blue pill thrice daily, but on the 18th, this was changed for two grains of calomel and half a grain of opium. Under this treatment the gums soon became slightly affected, and the tumour diminished in size. On the 1st of May, the fluctuating swelling at the upper part of the tumour was punctured, and about a table-spoonful of fluid evacuated. On the 7th May he was so far improved as to be made an out-patient.

This case shews that even at the "ninth hour" mercury has a very powerful effect on these chronic enlargements of the testicle. The disease had existed for many months, and it was feared at first that the structure of the organ was too far altered, to be benefited by any remedial means. The event fortunately proved otherwise.

---

### 83. CALOMEL AND OPIUM IN INFLAMMATION.

Dr. Yeats lately read a paper on this combination, at the Royal College of Physicians, by which it appears that this observant physician was among the first practitioners in this country who endeavoured to introduce the above remedy into general use as an important auxiliary—in many cases a substitute, for venesection. How long it is since mercury was first employed in the East Indies for the cure of hepatitis, we cannot pretend to say. The first printed account of the remedy that we have been able to obtain is given in the third volume of the Medical Museum (1764) by a surgeon from India; and the next account is by the late Dr. Hamilton of Lynn Regis, who published a paper in Duncan's Medical Commentaries for 1785. Dr. H. informs us that, at the close of the year 1764, the fleet which returned from the East Indies, "brought a worthy surgeon of the navy to England, who had served in that country for eight years." "By this gentleman I was informed that the established method of curing the hepatitis, or endemical inflammation of the liver, incident more particularly to Europeans, was by mercury."* After describing the method employed in India, Dr. Hamilton goes on to inform us that he tried the same remedy in the bilious autumnal fevers prevailing about Lynn, and found it very successful, especially when combined with opium. This success led him to an extension of the remedy. "The efficacy of mercury, says he, in ophthalmia, had long been established." "I considered that the general cause (be what it may) of an inflammatory diathesis, must be the same, whether the inflammation is seated in the meninges, pleura, lungs, liver, diaphragm, or any other internal part; and therefore the circumstance of the locality could make little or no alteration in the general intention of cure." This reasoning he forthwith applied to practice—and peripneumony being the first disease that fell under his care, after this resolution was taken, so it was the first species of inflammation in which calomel and opium was administered. The reader is aware that Dr. H. met with great encouragement.

"I was successful in a great number of cases, and under a variety of circumstances. I have had the satisfaction to see women far advanced in pregnancy, in a manner rescued from death, in the last stage of the peripneumony, by calomel and opium, after every other means,

---

* This refers to the account in the Medical Museum.

which had been tried, had failed in relieving the patients. I had the pleasure afterwards of seeing them go their full time, be safely delivered of living children, and enjoy the happiness of bearing several others since that period. I have known many a life saved in the symptomatic, variolous and morbillous peripneumony, by these medicines; and I never saw any remedies afford so certain and speedy relief in obstinate dry catarrhous coughs, as those, particularly when continued until the mouth became affected by the mercury. The same means have proved equally efficacious in pleurisies. But the most extraordinary and early relief I ever saw calomel and opium give, was in the phrenitis and paraphrenitis, which has been repeatedly experienced in a great number of cases. Inflammations of the intestines, and other parts within the abdomen, have most readily yielded to this treatment."

In puerperal fever, angina, acute rheumatism, gouty inflammation, &c. Dr. Hamilton derived great assistance from the combination in question. His plan was, first to take blood, "in quantity proportioned to the violence of the inflammatory symptoms, and the age and constitution of the patient." Then he cleared the bowels, and afterwards administered the calomel and opium till the mouth became sore, or the symptoms yielded, occasionally interposing general or local bleeding, if the disease was obstinate.

Several writers, among whom we need only mention Lind and Clark, afterwards gave their testimony towards the efficacy of calomel and opium in certain inflammatory complaints. And then comes Dr. Yeats, who, in the year 1802, published a paper in the 7th volume of Duncan's Annals of Medicine, on the same subject. Dr. Yeats informed the audience that he was led to the use of this remedy by chance, and not by design. He gave a dose of calomel to a patient labouring under ophthalmia, intending the next day to have the vessels of the conjunctiva divided. The medicine instead of purging, salivated the patient, and cured the inflammation. Dr. Y. then extended the practice to other inflammations—and, as far as we can perceive, precisely under the same rules and restrictions as Dr. Hamilton laid down so long before.

At the close of the paper, Dr. Yeats made some judicious observations on the danger of exhibiting mercury in scrofulous and bad constitutions, lest the remedy should prove worse than the disease.

We have not the smallest doubt that the medicine under consideration, would prove equally as effectual in other inflammations as in those of the eye, the liver, and the heart. But we are satisfied that this remedy will never come into general use for inflammation of the lungs, pleura, and many other internal parts, for two reasons:—first, because the inflammations of these structures are not very suddenly fatal, generally speaking:—secondly, because they are, for the most part, curable by the other antiphlogistic means, without the risk of salivation. When Dr. Yeats or any other man tells us to go on with mercury till we gently affect the mouth, but never to induce salivation, we wonder that clinical observation did not teach them how difficult this rule of practice too often is. How frequently do we find that mercury, when pushed on briskly, as it must be for the cure of an internal inflammation, runs to the mouth and produces violent salivation in spite of us? When giving mercury slowly, for a syphilitic or chronic complaint, we have some control; but in acute diseases, if we give it with the view of exciting constitutional effects, we must lay out our accounts for *salivation*, whatever care we take. This event, which all practical men are aware of, will prevent, as we said before, the remedy from coming into general use. But where the eye, for example, is deeply inflamed, and vision is endangered, then men will run the risk of salivating rather than bleeding their patients. So, in the East Indies, if acute hepatitis does not give way readily to antiphlogistics, practitioners are aware that suppuration quickly ensues, and therefore they give mercury at the risk of even profuse ptyalism. In inflammation of the heart, who would balance between death and salivation? But considering the outcry that is made against mercury, both by medical men and the public, we do not wonder that practitioners should only have recourse to an active administration of that remedy, when the life or some important function of their patient is at stake.

### 84. New Method of treating Amaurosis.

M. Majendie having previously proved by experiment that the concurrence of the nerves of the fifth pair, are as necessary to vision as that of the optic nerves, supposed that amaurosis might sometimes arise from a paralysis of the first of these nerves, and that by powerfully exciting them, we might produce advantageous results. Being satisfied that these nerves might be punctured with impunity, and having remarked that the pupil was contracted whenever he pricked either of the orbitar branches of the fifth pair, he passed one needle into the frontal nerve, and another into the superior maxillary, and connected each with one of the poles of a voltaic pile. In a case of amaurosis of one-half of the retina, accompanied with paralysis of the superior eyelid and some of the muscles of the eye, in three months the retina and muscles of the eye were restored to the performance of their healthy functions.—*Journal des Progrès, Vol.* IV.

---

### 85. Signs of the Times—Brightening Prospects.

Non semper imbres nubibus hispidos
Manant in agros; aut mare caspium
Vexant inæquales procellæ
Usque.

It was but a very short time before the downfall of Napoleon that men began to think the war between France and England interminable—at least, on any other principle than that of extermination. "*Delenda est Carthago*" was the motto! Yet a premature fall of snow—a depression of the mercury in the thermometer, totally changed the scene—the voice of war was hushed—Bellona no longer thundered on the field—the Gallic Cock and British Lion embraced—and Europe was in profound repose! So again, it is only a few short months, we might say weeks, since the profession of physic in this country, exhibited a scene of anarchy, animosity and intestine war, that threatened a dissolution of all those bonds and ties by which medical society is held together. Now, how changed is the scene! The stormy passions are allayed—the lion is seen feeding with the lamb—the vulture and the dove fly peaceably together—scandal has become a drug—abuse is out of fashion—and—however incredible it may appear—a certain weekly Journal is as decent as it is dull! To crown the whole, we understand that a once celebrated orator and reformer means to call a meeting of the profession at the Free-mason's Tavern, in order to celebrate, with due libations, the return of peace, amity, and liberality among all ranks of medical society!

Hic dies, anno redeunte, festus
Corticem astrictum pico dimovebit
Amphoræ fumum bibere institutæ
Consule Tullo.
Sume, Mæcenas, *cyathos* amici
Sospitis *centum*; et vigiles lucernas
Profer in lucem:—*procul omnis esto*
*Clamor et ira*.

It might not be uninteresting to trace the causes of this *apparently* sudden revolution in the minds of men. These causes have been long in operation, though their effects have only recently become conspicuous. One great cause is the loss of that attraction which led on vituperation to the summit of success. No passion of the human mind, however strong, and however deeply rooted in our nature, can bear constant and long-continued excitement. It must become sated, and disgust soon follows. Happily for the peace and prosperity of medical society, the attraction of scandal, like the attraction of the moon, has passed the zenith, and ceased to raise the tide of evil passions. Attraction withdrawn, gravitation necessarily comes into play, and the tide will roll back to its source with much greater velocity than it advanced.

It becomes the bounden duty—and not less the duty than the interest, of every medical man, to foster and promote this return of amity and peace in a liberal profession, by cautiously abstaining from every act or expression that can engender bad feeling—and by leaving the agitators to their own precipitate and natural descent into the ocean of oblivion. No other impetus is necessary than the gravity which is inseparable from evil deeds and dark designs.

---
